# E-Module Learning for Scaling Serious Illness Communication Skills Teaching: A Pilot Study in Family Medicine and Palliative Care

**DOI:** 10.1089/pmr.2024.0048

**Published:** 2024-12-26

**Authors:** Helen James, Paul Krueger, Daphna Grossman, Warren Lewin

**Affiliations:** ^1^ Division of Palliative Care, University Health Network, Toronto, Ontario, Canada.; ^2^ Department of Family and Community Medicine, University of Toronto, Toronto, Ontario, Canada.; ^3^ Division of Palliative and Supportive Care, North York General Hospital, Toronto, Ontario, Canada.

**Keywords:** communication skills, e-modules, learner/faculty perceptions, serious illness communication

## Abstract

**Background:**

Serious illness communication (SIC) competency is essential for health care professionals. However, many clinicians receive little-to-no SIC training, and there is little evidence as to which teaching method is most feasible to incorporate into postgraduate curricula. Two e-modules were created to adapt high-yield knowledge to deliver asynchronous, time-efficient, standardized communication skills teaching. This project evaluated SIC e-module teaching feasibility, learner and faculty perceptions toward e-module learning on this topic, as well as learner confidence and skill usage post-completion.

**Methods:**

Family Medicine residents and palliative care fellows from two training sites were invited to asynchronously complete the e-modules on their own time and complete a survey to assess attitudes, perceptions, and needs toward them and impact on SIC skills immediately and 1-month post-completion. Faculty from the main site were also invited to view the e-modules and complete a survey immediately afterward assessing attitudes, perceptions, and feasibility on SIC e-module learning.

**Results:**

In total, 19/50 (38%) learners completed the e-modules and post-training survey and 14/19 (73%) of those learners completed the 1-month follow-up survey. In total, 13/60 (22%) faculty completed the survey. Participants liked the structure and design of the e-modules and felt they were appropriate for their learners’ level of training, were effective, time-efficient, and provided relevant SIC information. Case-based video demonstrations were identified as the most useful teaching method. Most learners intended to use new skills in clinical practice, rewatched both e-modules within 1 month of initial viewing, and reported using learned skills in practice.

**Conclusion:**

E-module training provides a standardized method to scale postgraduate SIC skills teaching asynchronously and was well liked by learners and faculty. Barriers exist to completing them outside of a core curriculum. Early data suggest e-modules can be used iteratively and further research is needed to determine how their use impacts communication confidence and competency.

## Background

Honest and empathic serious illness communication (SIC) skills are essential for the delivery of high-quality health care, particularly for clinicians leading conversations with patients facing serious illness. Emerging literature points to structured, skill-based training resulting in improved serious illness conversations.^1–4^ Postgraduate medical education training programs expect their residents to have a level of communication skill competency; however, there is no standard to teach them.^5–7^ Additionally, many clinician teachers have completed neither skills training themselves^8^ nor faculty development to build their teaching skills.^9^

To our knowledge, there is no stand-alone standardized SIC skills training program widely used across Canada. Two well-known North American evidence-based SIC training programs, VitalTalk^10^ and Serious Illness Care Program,^11^ have been combined, adapted, and taught at our institution. To rapidly scale SIC training at our institution, respond to requests for an asynchronous learning option, and provide faculty with an off-the-shelf teaching tool for foundational SIC skills teaching, we turned to e-learning.

E-learning can increase medical knowledge,^12^ is at least equivalent to in-person teaching,^13^ and is acceptable to undergraduate^14^ and postgraduate^15^ learners. E-modules allow students to interact with material in a safe space and allow for asynchronous preparatory learning to increase time learners and preceptors can spend together honing skills through observed practice at the bedside or workshops, which is a cornerstone of SIC training.^16^

A literature review and local needs assessment informed the creation of two e-modules that combined and adapted core knowledge from the aforementioned programs with an aim to deliver standardized SIC skills learning in a safe, portable, and time-efficient manner.

One e-module introduces a structured guide to lead serious illness conversations that was adapted from Ariadne Lab’s Serious Illness Conversation Guide and the REMAP mnemonic.^17^ It comprises an introductory check-in, a description of the purpose for the conversation, an exploration of what the patient/family knows prior to validating/relaying/reframing serious news, a pause to acknowledge and respond to emotion, an exploration of values and goals, and an align statement before making a value-concordant plan and closes with gratitude and an explicit statement of when follow-up will occur next. The guide is supplemented by evidence supporting it, outlines key principles to maximize its use in practice, and is presented using interactive methods and video demonstrations using noncancer and cancer case examples that were created by the authors. An approach to specifically cultivating illness understanding and prognostic uncertainty is described^18^ and has an accompanying video demonstration. The module is divided into four parts: background evidence and rationale for using a conversation guide, preparing for the conversation, having the conversation, and documenting the conversation.

Another e-module introduces and names practical, discrete, evidence-informed communication skills including sharing serious news using a headline or reframe statement; acknowledging and responding to emotions using wish, worry, and NURSE statements; and asking permission to encourage shared decision making and to afford patient control, which are described in Arnold et al.^19^ A description, rationale, and interactive demonstration using flip cards, knowledge test questions, and video demonstrations between a clinician and a standardized patient simulating clinical scenarios are used to teach each skill.

Each e-module can be completed asynchronously, independently, and in any order online.

## Objective

This pilot project aimed to evaluate postgraduate and faculty perceptions of the SIC e-module learning experience as well to explore the feasibility of using e-modules for teaching about SIC. It also aimed to explore the impact of SIC e-module training on learner confidence and perceived skill usage post-completion.

## Methods

### Design

One of the study authors developed the e-modules using Articulate 360 software. They were made accessible across desktop, tablet, and mobile devices and were password protected.

Family Medicine postgraduate learners from two training sites at the University of Toronto were invited by their Program Directors to complete both e-modules asynchronously and to voluntarily complete online surveys immediately afterward and 1 month later. Palliative Medicine Fellows from the main study site were also invited by their Program Director to complete the same process. The first survey gathered demographic information, attitudes toward e-learning, as well as the content and design of the specific e-module and perceptions and needs for SIC e-module learning. The follow-up survey gauged ongoing use of the e-modules and skills taught in them, as well as confidence to engage in serious illness conversations.

Family Medicine and Palliative Care faculty from one of the study sites were invited by either the Chief of Family Medicine or the Palliative Care Division Head to complete the e-modules and a survey immediately afterward. The survey gathered demographic information and participants’ perceptions of the e-modules’ usability and relevance in supporting SIC skill development for their learners. All respondents received a gift card to a local retailer as compensation for study participation. Survey responses remained anonymous.

To our knowledge, no validated tool to assess perceptions and attitudes toward SIC e-module learning exists, so the authors created one for this project distributed online using Qualtrics^XM^. Consent to voluntarily participate in the study was described on the first page of the survey and consent was implied by a participant completing the survey. To assess feasibility, most questions used 5-point Likert scales with agreement ratings from “strongly disagree” to “strongly agree” and usefulness ratings ranging from “not at all useful” to “very useful.” Participants were additionally asked to identify their top two most effective e-module teaching methods from a list that included video demonstration, information tabs, drop-down menus, knowledge-check questions, flash cards, and a matching exercise. Free-text questions allowed participants to describe why they preferred various teaching methods within the e-modules as well as asked them to suggest recommendations, if any, for improvement. Postgraduate participants were asked to list any skills they recalled learning about from the e-modules immediately and 1 month after completing the e-modules. Participants were also asked 1 month post e-module completion to self-report on their confidence toward skill usage and whether they accessed the e-modules again and used the knowledge and skills in clinical practice.

Research ethics board (REB) approval was granted by the University Health Network REB # 22-5986.

### Setting/subjects

The main study site is a large urban teaching hospital comprising 30 family medicine residents, and the satellite site is a community teaching hospital with 10 family medicine residents; each site includes a mandatory 1-month inpatient palliative care rotation as part of a 2-year training program. The residents at both sites are part of the largest family medicine training program in Canada. The main site also trains palliative care fellows in a 1-year subspeciality program in adult palliative medicine.

### Measurements

#### Data analysis

Five-point Likert scale data were collapsed into two categories (e.g., “strongly agree” and “agree” vs. “other”). Descriptive statistics (frequencies and percentages) were calculated and reported. The descriptive statistics of the responses from the family medicine residents were compared to the palliative care fellows’ responses; however, there were minimal differences seen. The faculty responses were not compared given the small sample size.

## Results

In total, 19 of 50 (38%) eligible learners completed the first survey, and 14 of those 19 (74%) completed the follow-up survey. Learner respondents included seven postgraduate year (PGY)1 family medicine residents, seven PGY2 family medicine residents, and five palliative care fellows. Thirteen of 60 (22%) faculty completed the e-modules and survey. Most faculty came from the palliative care department (*n* = 10, 77%). Most faculty (*n* = 10, 77%) reported having previously taught about SIC skills and 6 of 13 (46%) faculty reported having completed faculty development training related to teaching about SIC (Table 1).

**Table 1.** Demographic and Practice Characteristics of Learners and Faculty

Demographic and practice characteristics	Palliative care learners (*n* = 5) (*n*, %)	Family medicine learners (*n* = 14) (*n*, %)	Learners combined (*n* = 19) (*n*, %)	Faculty (*n*, %)
Age (learners *n* = 19)				
18–24	—	1 (7.1%)	1 (5.3%)	Not asked
25–34	2 (40%)	13 (92.8%)	15 (78.9%)	
35–44	3 (60%)	—	3 (15.8%)	
Gender (learners *n* = 19, faculty *n* = 13)				
Female	4 (80%)	10 (71.4%)	14 (73.7%)	11 (84.6%)
Male	1 (20%)	3 (21.4)	4 (21.1%)	2 (15.4%)
Genderfluid/Gender nonconforming	—	1 (7.1%)	1 (5.2%)	—
Year of training (learners *n* = 19)				
PGY 1	—	7 (50%)	7 (36.8%)	N/A
PGY 2	—	7 (50%)	7 (36.8%)	
PGY 3	5 (100%)	—	5 (26.4%)	
Number of years in practice (faculty *n* = 13)			N/A	
1–5	N/A	N/A		7 (53.8%)
6–15				4 (30.8%)
16–25				2 (15.4%)
Specialty (learners *n* = 19, faculty *n* = 13)				
Family medicine			14 (73.7%)	3 (23.1%)
Palliative care			5 (26.3%)	10 (76.9%)
Location of medical school (learners *n* = 19)				
Canada		13 (92.9%)	13 (68.4%)	Not asked
International	5 (100%)	1 (7.1%)	6 (31.6%)	

N/A, not applicable; PGY, postgraduate year.

### E-module usage for teaching and learning

Most learners (*n* = 15/19, 79%) and faculty (*n* = 9/13, 69%) completed both e-modules within 21–60 minutes. Most learners had been taught using e-modules “occasionally” (*n* = 8/19, 42%) or “often” (*n* = 9, 47%), whereas most faculty (*n* = 10/13, 77%) reported not routinely incorporating e-modules into their teaching.

### Attitudes and perceptions toward the e-modules’ content and design

Participants liked the structure and design of the e-modules and felt they were appropriate for their/their learners’ level of training, were effective and time-efficient, and provided relevant SIC information (Table 2). Learners and faculty both found the video demonstrations of skills in practice (*n* = 16/19, 84% and *n* = 10/13, 77%, respectively) to be the most effective teaching method within the e-modules as they could see the skills in action, and it reinforced the knowledge presented in the e-modules. After completing the e-modules, all learners (*n* = 15/15, 100%) planned to apply the knowledge, and most (*n* = 13/15, 87%) intended to use the language in clinical practice.

**Table 2.** Learner and Faculty Perceptions of the Content and Format of the E-Modules

Perceptions of the content and format	Agree or strongly agree (*N*, %)			
	PC learners (*N* = 5)	FM learners (*N* = 14)	Learners combined (*N* = 19)	Faculty (*N* = 13)
The e-module’s content met the learning objectives.	5 (100%)	14 (100%)	19 (100%)	12 (92.3%)
The e-modules delivered content at an appropriate level for my/my learners learning.	4 (80%)	14 (100%)	18 (94.7%)	13 (100%)
I would recommend the e-modules to a colleague.	5 (100%)	11 (76.6%)	16 (84.2%)	13 (100%)
I am interested in using these e-modules with my learners.	Not asked	Not asked	Not asked	12 (92.3%)
The order of the content was presented in an effective way.	5 (100%)	14 (100%)	19 (100%)	13 (100%)
The e-modules were easy to navigate.	5 (100%)	14 (100%)	19 (100%)	Not asked
The e-modules provided information relevant to my medical practice/my learners.	5 (100%)	13 (92.8%)	18 (94.7%)	13 (100%)
The e-modules were a valuable use of my time.	5 (100%)	12 (85.7%)	17 (89.5%)	Not asked
The e-modules were a time-efficient way of learning.	5 (100%)	12 (85.7%)	17 (89.5%)	Not asked

FM, family medicine; PC, palliative care.

### Confidence after watching the e-modules and 1 month later

Most learners felt more confident in their ability to empathically support their patients (*n* = 15/19, 79%) and to lead serious illness conversations (*n* = 17/19, 90%) after completing the e-modules, and there did not appear to be a decline in confidence 1 month later (*n* = 12/14, 86% and *n* = 11/14, 79%, respectively). Over half of the learners revisited both e-modules within 1 month of initially completing them (*n* = 8/14, 57%) and reported using both the communication skills (*n* = 12/14, 86%) and the structured guide (*n* = 11/14, 79%) in practice.

### Incorporating the e-modules into a communication skills curriculum

Learners and faculty agreed the e-modules taught both core and advanced communication skills well and could be used to supplement didactic and bedside teaching (Table 3).

**Table 3.** Learner and Faculty Perceptions Toward How the E-Modules Can Be Incorporated

Perceptions toward how the e-modules can be incorporated	Agree or strongly agree (*N*, %)			
	PC learners (*N* = 5)	FM learners (*N* = 14)	Learners combined (*N* = 19)	Faculty (*N* = 13)
The e-modules provided valuable core communication skills training.	5 (100%)	12 (85.7%)	17 (89.5%)	Not asked
The e-modules provided valuable advanced communication skills training.	5 (100%)	12 (85.7%)	17 (89.5%)	Not asked
The e-modules taught core communication skills well.	Not asked	Not asked	Not asked	13 (100%)
The e-modules taught advanced communication skills well.	Not asked	Not asked	Not asked	11 (84.6%)
The e-modules could be an additional teaching method for core communication skills (e.g., on top of didactic lecture or other methods).	5 (100%)	12 (85.7%)	17 (89.5%)	13 (100%)
The e-modules could replace a didactic lecture on core communication skills.	1 (20%)	7 (50%)	8 (42.1%)	6 (46.2%)
The video demonstrations were an acceptable additional teaching method to an in-person observation of a preceptor talking to a patient at the bedside.	5 (100%)	13 (92.8%)	18 (94.7%)	13 (100%)
The video demonstrations were an acceptable substitute teaching method to an in-person observation of a preceptor talking to a patient at the bedside.	1 (20%)	8 (57.1%)	9 (47.3%)	Not asked
The video demonstrations would be helpful to watch prior to practicing the skills at the bedside with a real patient.	5 (100%)	12 (85.7%)	17 (89.5%)	Not asked

## Discussion

This study evaluated the feasibility of using asynchronous e-modules to teach about SIC skills drawn from two popular evidence-backed North American SIC training programs. The e-modules were designed to be efficient and scaled across an organization. Pilot postgraduate and faculty data indicate support for integrating e-module learning to teach on this topic. The e-modules were associated with increased learner confidence in supporting patients with empathy and leading serious illness conversations. The learners reported using the knowledge within a month after completion and showed a trend toward confidence being maintained over time. Barriers to asynchronous training were theorized that can inform future research on postgraduate e-module learning.

Learning about communication skills can be done similarly to learning other procedural skills.^20,21^ The component tasks of learning these skills include expert observation, deliberate practice, and observed feedback.^22^ Participants in this study reported video demonstrations as the most useful learning method, which provides expert observational opportunities as part of the procedural skill learning sequence. Viewing video demonstrations prior to attempting skills has been shown to improve performance and learning,^23–25^ which may be due to a video’s ability to accurately reflect real-life presentations.^26^ Asynchronous viewing of foundational SIC skills in the e-modules may increase time during synchronous teaching blocks for focused, observed deliberate practice of SIC skills.

Factors leading to effective long-term learning are repetitive, active, and standardized learning opportunities.^26^ E-module teaching can provide iterative and standardized learning opportunities needed to support learning. More than half of the learners in our study reported using the skills in practice within a month of completing the e-modules. Surprisingly, many also made time to voluntarily revisit the e-modules on their own during the month after completing them. Learners value e-module learning, especially when paired with facilitator-led discussions, simulation, or bedside teaching,^27^ which is encouraging as it supports our work in building upon existing strong SIC teaching programs that use such multimodal learning.

A strength of the teaching design of the e-modules is their intentional separation of content into two parts; one e-module focusing on discrete communication skills that support conversation effectiveness and another e-module focusing on the structure and flow of a fulsome serious illness conversation. Teaching discrete communication skills allows learners to understand the components of the “procedure” more readily^28^ and assists both learners and educators to identify areas for communication skill development that can be taught and honed as part of teaching about communication related to serious illness or other aspects of health care. This is consistent with the process of learning a procedure in that knowledge acquisition of component skills is required to master the overall task.^29^

Despite the e-modules being easily accessible online and time-efficient and covering core postgraduate learning competencies, barriers to e-module completion are known^30^ and may help to explain the study’s low response rates. Protecting core curriculum time to teach about communication may bolster participation and importantly contribute to closing a long-standing gap in teaching on this topic. Future research should investigate how to effectively integrate e-module learning into a SIC curriculum. Moreover, once the e-modules are created, the content can be efficiently customized online and scaled for different specialty and health professions use. Given that many faculty in this study reported not having completed a SIC-related faculty development course, e-modules can provide evidence-based standardization to teach about a complex topic using a common language across specialties. Finally, e-modules can be accessed on various devices providing a just-in-time learning option that may help build competency, which should be the focus of future research.

### Limitations

This study has several limitations. The survey response rates were low, and our sample came from two training sites within the same city, which may limit generalizability. Participants were asked to complete the e-modules and surveys in their own time just after completing board examinations and during a busy time in the academic year that may have made it difficult to add a voluntary learning opportunity into busy schedules. The decision to protect time or not for e-module completion is an important decision and needs to consider the burdens already placed on learners and faculty within and outside of routine work hours. Moreover, participation in the study was voluntary, potentially contributing to self-selection bias of those already interested in advancing their communication skills further impacting the generalizability of the data. Longitudinal follow-up was limited to a 1-month time frame that did not measure objective knowledge and skill, which future studies should investigate.

## Conclusion

Communication is a key competency required for health care providers and yet is infrequently taught. E-modules provide standardized, scalable, and iterative opportunities to learn about foundational evidence-based SIC skills. They provide a ready-made teaching tool with a common language for faculty to use who haven’t been trained to teach about communication skills. Pilot data suggests that e-modules are promising for adapting and widely distributing foundational skills from established curricula. However, implementation barriers to e-module learning should be explored.

## Abbreviations Used

PGY: Postgraduate year
REB: Research Ethics Board
SIC: Serious illness communication
